# Prevalence of high, medium and low-risk medical conditions for pneumococcal vaccination in Catalonian middle-aged and older adults: a population-based study

**DOI:** 10.1186/s12889-017-4529-8

**Published:** 2017-06-29

**Authors:** O. Ochoa-Gondar, I. Hospital, A. Vila-Corcoles, M. Aragon, M. Jariod, C. de Diego, E. Satue, EPIVAC Study Group

**Affiliations:** 1 Primary Health Care Service “Camp de Tarragona”, Institut Catala de la Salut, Rambla Nova 124, D,1°A, 43001 Tarragona, Spain; 2 Unitat de Suport a la Recerca of Tarragona, Institut Universitari d’Investigació en Atenció Primària Jordi Gol (IDIAP Jordi Gol), Tarragona, Spain; 3 Information System for the Improvement of Research in Primary Care (SIDIAP), Primary Care Research Institute Jordi Gol, Universitat Autonoma de Barcelona, Barcelona, Spain; 4 Information Systems Department, Hospital Joan XXIII, Tarragona, Spain

**Keywords:** Adults, Chronic illness, Pneumococcal disease, Prevalence, Streptococcus Pneumoniae, Risk factor

## Abstract

**Background:**

Updated population-based data on the frequency and distribution of risk factors for pneumococcal disease is scarce. This study investigated the prevalence of distinct comorbidities and underlying risk conditions related to an increasing risk of pneumococcal disease among Catalonian middle-aged and older adults.

**Methods:**

Cross-sectional population-based study including 2,033,465 individuals aged 50 years or older registered at 01/01/2015 in the Catalonian Health Institute (Catalonia, Spain). The clinical research database of the *Information System for the Development of Research in Primary Care (SIDIAP database)* was used to identify high-risk (asplenia and/or immunocompromising conditions) and other increased-risk conditions (chronic pulmonary, cardiac or liver disease, diabetes mellitus, alcoholism and/or smoking) among study subjects.

**Results:**

Globally, 980,310 (48.2%) of the 2,033,465 study population had at least one risk condition of suffering pneumococcal disease (55.4% in men vs 42.0% in women, *p* < 0.001; 41.7% in people 50–64 years vs 54.7% in persons 65 years or older, *p* < 0.001). An amount of 176,600 individuals (8.7%) had high-risk conditions (basically immunocompromising conditions). On the other hand, 803,710 persons (39.5%) had one or more other risk conditions. In fact, 212,255 (10.4%) had chronic pulmonary diseases, 248,377 (12.2%) cardiac disease, 41,734 (2.1%) liver disease, 341,535 (16.8%) diabetes mellitus, 58,781 (2.9%) alcoholism and 317,558 (15.6%) were smokers.

**Conclusion:**

In our setting, approximately 50 % of overall persons 50 years or older may be considered at-risk population for pneumococcal disease (almost 10 % have high-risk conditions and 40 % have other risk conditions).

## Background

*Streptococcus pneumoniae* is a Gram-positive bacterium that typically colonises the respiratory tract in humans, being the cause of serious illness and death in some individuals. After colonisation, possible outcomes are the clearance, the asymptomatic persistence (carrier state), or the bacterium can progress to the disease, causing mucosal infections (otitis, sinusitis, bronchitis and nonbacteremic pneumonias) or resulting in invasive pneumococcal disease (IPD) such as bacteremic pneumonias, meningitis or sepsis. The outcome depends on interactions between factors related to the host, therapy and microorganism. [1, 2].

At present, pneumococcal infections remain a major cause of morbidity and mortality around the world, being one of the ten leading causes of death worldwide. [3] The risk for pneumococcal infection has been described as “high” in persons with immunosuppressive conditions, organ or bone marrow transplantation, immunosuppressive therapy and chronic renal failure or nephrotic syndrome as they have decreased responsiveness to polysaccharide antigens or an increased rate of decline in serum antibody concentrations. [4] Moreover, persons with functional or anatomic asplenia are at the highest risk for pneumococcal infection, because this condition leads to reduced clearance of encapsulated bacteria from the bloodstream. [1, 2, 4] Other adults at increased risk for developing pneumococcal infection or experiencing severe disease and complications include immunocompetent persons with chronic heart diseases, chronic pulmonary diseases, chronic liver diseases, diabetes mellitus, alcoholism and smoking. [5–8] Elderly persons (i.e, 65 years or older) are a major high-risk group for pneumococcal infections considering that certain degree of immunosenescence occurs in the elderly and, furthermore, the prevalence of chronic illnesses increases with age.

Several studies have investigated the prevalence of distinct risk conditions to suffer IPD among adult populations and have reported incidence of IPD among healthy adults as compared with those with underlying conditions. [8–11] Usually, the published data was calculated for distinct children age subgroups, adults 18–64 years and elderly persons, but generally there are no specific data disaggregated for distinct adult age subgroups (i.e, younger or middle-aged adults) despite the fact they may have distinct risks for pneumococcal disease. According to these studies, incidence of IPD is around 10 cases per 100,000 population-year among healthy adults over 18 years, with rates up to 50-times higher in some immunocompromised individuals (e.g, haematological cancer and/or HIV infection). [8, 11] According to published data, 39% of USA adults over 18 years have some risk condition for pneumococcal disease whereas this proportion ranges between 13 and 67% in European studies. [8–11].

At present, two pneumococcal vaccines are available for use in adults: the “classical” 23-valent pneumococcal polysaccharide vaccine (PPV23) and the “new” 13-valent protein-polysaccharide conjugate vaccine (PCV13). The PPV23 was marketed in 1983 [4] whereas the PCV13 has only been available for use in adults since 2012. [12] From a public health point of view, the availability of two different vaccines for adults and the possibility of sequential vaccination with both vaccines is a key point to establish different vaccination strategies.

In Catalonia and Spain, since the 2000s, the PPV23 is recommended and publicly funded for all people aged 65 years or older (with or without risk conditions) as well as for those persons 18–64 years with certain underlying risk conditions such as asplenia, immunocompromising conditions, chronic pulmonary, cardiac, renal or liver disease, diabetes, alcoholism and smoking. [13, 14] The PCV13 is publicly funded only for “high” risk individuals (i.e, immunocompromised patients, anatomical or functional asplenia, cochlear implants and CSF leaks). [13, 14] The PCV13 is also prescribed by some doctor for some immunocompetent patients with other risk conditions (e.g. chronic bronchitis, asthma and/or cardiac disease) although it is not publicly funded in these individuals. [15].

In 2015, the reported pneumococcal vaccination coverage in Catalonia was 39% among adults ≥50 years-old (4.8% in 50–59 years vs 35.5% in 60–69 years vs 71.9% in 70–79 years vs 79.5% in 80 years or older). [16] According to the most recent published data, incidence of IPD in Catalonia during the 2012–2014 period was 12.3 cases per 100,000 all-age population and year (40.9 in children under 2 years, 22.3 in children 2–4 years, 3.9 in 5–19 years, 7.4 in 20–64 years and 31.8 in people ≥65 years). [17].

As mentioned above, several studies have reported the prevalence of chronic illnesses and underlying conditions among hospitalised patients with IPD or pneumonia, [8–11] but there is limited population-based data reporting the prevalence of some conditions (especially low prevalence conditions) classically related with increasing risk for pneumococcal disease. Accurate prevalence data about risk conditions for pneumococcal disease is needed to calculate the true magnitude of at-risk groups and estimate the size of different target populations to implement distinct alternative antipneumococcal vaccination strategies currently available. [13–15, 18–20].

The present study investigated the prevalence of distinct comorbidities and underlying conditions related to increasing risk (high-, medium- and low-risk) for pneumococcal infections among Catalonian middle-aged and older adults. High-risk persons in the same population have been previously described elsewhere. [21].

## Methods

This is a cross-sectional population-based study involving all individuals aged 50 years or older, who were registered in the Primary Health Care Centres (PHCCs) of the *Catalonian Health Institute* on January 1, 2015 (*N* = 2,033,465 persons).

In Catalonia, a region in North Eastern Spain with seven million people, there are 358 PHCCs (comprised of family physicians, nurses and support staff) which are distributed by geographical area and are responsible for the health care of the population in their areas. The *Catalonian Health Institute* manages 274 PHCCs, serving a population of approximately five million people. Doctors and nurses systematically use electronic medical records to record medical diagnoses, underlying conditions, prescriptions, and other clinical patient management activities coded according to the International Classification of Diseases, 10th Revision (ICD-10).

The *Catalonian Health Institute Information System for the Development of Research in Primary Care* (“SIDIAP” database) compiles coded clinical information from the Electronic PHCC’s records, [22] and it has been used as the data source for this report. Quality criteria for clinical data of the SIDIAP research database were reported in a validated comparison process. [23] The SIDIAP sample is representative of the general Catalonian population in terms of geography, age and sex distributions, according to the official census. [22].

The “SIDIAP” research database was used to identify chronic comorbidities and underlying medical conditions among study subjects, who were classified into three risk strata on the basis of immunocompromise degree and current recommendations for pneumococcal vaccination. [4, 13, 14] Risk stratum 1 (highest-risk) included persons with anatomic or functional asplenia (ICD-10 codes: D57,D73, Q89), cochlear implants (Z96.2, Z45.3), CSF leaks (Z98.2), primary immunodeficiency (D80-D84), HIV infection (B20-B24), nephrotic syndrome (N04, N39.1), severe chronic renal failure (N18-N19 with glomerular filtration rate ≤ 30 ml/min), bone marrow transplantation (Z94), solid organ or haematological neoplasia (C00 to C97) diagnosed within previous 5 years, long-term immunosuppressive medication and/or radiotherapy in the previous 12 months (coded according to specific SIDIAP codes). Risk stratum 2 (medium-risk) included immunocompetent patients without a level 1 condition but who had a history of chronic pulmonary/respiratory disease (including chronic bronchitis/emphysema [J41-J44], asthma [J45-J46] and/or other chronic pulmonary diseases [P27, E84, J47]), chronic cardiac disease (including congestive heart failure [I50], coronary artery disease [I20-I22, I25] and/or other chronic heart diseases [I05-I08, I11,I35-I37,I42, I51.7]), chronic liver disease (including chronic viral hepatitis [B18], cirrhosis [K74] and/or alcoholic hepatitis [K70]), diabetes mellitus [E10-E14], alcoholism [F10, G31.2, G62.1, G72.1, I42.6, K29.2, K70] and smoking [F17]. Risk stratum 3 (lowest risk) included immunocompetent persons without level 1 or 2 conditions. We assumed that information in the *SIDIAP* database was complete, so a condition was considered absent if it was not recorded.

The statistical differences between prevalence for distinct risk conditions according to distinct population subgroups were evaluated using the Chi-squared tests, or Fisher’s exact test as appropriate. Odds Ratios (ORs) with 95% confidence intervals (CIs) were estimated to compare prevalence between gender and age groups. Statistical significance was set at *p* < 0.05 (two-tailed). Data were analyzed using the SPSS version 18.0 (SPSS Inc., Chicago, IL, USA).

## Results

Of the total 2,033,465 study population, 935,705 (46%) were men and 1,097,760 (54%) were women, being 1,021,648 (50.2%) aged 50–64 years and 1,011,817 (49.8%) aged 65 years or older.

**Fig. 1:**
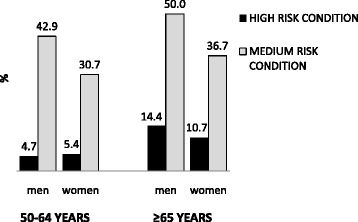
Prevalence of middle-aged and older adults with high- or medium-risk condition to suffer pneumococcal disease in Catalonia, Spain

Globally, 980,310 (48.2%) of the 2,033,465 study population had at least one risk condition to suffer pneumococcal disease (55.4% in men vs 42.0% in women, *p* < 0.001; 41.7% in people 50–64 years vs 54.7% in persons 65 years or older, *p* < 0.001). Among the 50–64 years age subgroup, prevalence of persons with any high-risk condition (stratum 1) was 4.7% in men and 5.4% in women, whereas prevalence of persons with any medium-risk condition (risk stratum 2) was 42.9% in men and 30.7% in women. Among the 65 years or older age subgroup, prevalence of persons with any high-risk condition (stratum 1) was 14.4% in men and 10.7% in women, whereas prevalence of persons with any medium-risk condition (risk stratum 2) was 50% in men and 36.7% in women (Figure 1).

Overall, 649,014 (31.9%) of the total 2,033,465 study subjects had only one risk condition, 243,127 (12.0%) had two risk conditions, 69,308 (3.4% had three risk conditions and 18,861 (0.9%) had four or more risk conditions.

If we consider those conditions associated with highest risk for pneumococcal infections (risk stratum 1), 3274 (<0.1%) had primary immunodeficiency, 589 (0.2%) HIV infection, 1480 (0.1%) nephrotic syndrome, 15,468 (0.8%) had severe renal failure, 5034 (0.2%) had received bone marrow transplantation, 103,948 persons (5.1%) had recent cancer diagnosis and 72,040 (3.5%) received immunosuppressive treatment/radiotherapy in last 12 months. An amount of 294 (<0.1%) had asplenia, 76 (<0.1%) cochlear implants and 41 (<0.1%) CSF leaks.

Considering that some persons had more than one high-risk condition, only 176,600 individuals (8.7%) of the overall study population were classified in risk stratum 1 (9.2% in men vs 8.2% in women, *p* < 0.001; 5.1% in persons 50–64 years vs 12.3% in persons 65 years or older, *p* < 0.001).

If we consider the presence of other medical conditions related to an increased risk of pneumococcal infections classified in risk stratum 2, 212,255 persons (10.4%) had chronic pulmonary/respiratory diseases, 248,377 persons (12.2%) had chronic heart diseases, 41,734 (2.1%) chronic liver diseases, 341,535 (16.8%) diabetes mellitus, 58,781 (2.9%) alcoholism and 317,558 (15.6%) were smokers.

Overall, 803,710 individuals (39.5%) of the 2,033,465 study subjects were classified in risk stratum 2 because they had one of the above mentioned conditions but had no concomitantly conditions mentioned in risk stratum 1 (46.2% in men vs 33.8% in women, *p* < 0.001; 36.7% in persons 50–64 years vs 42.4% in persons 65 years or older, *p* < 0.001).

A total of 1,053,155 persons (51.8%) of the overall study population were classified as risk stratum 3 (lowest risk) because they did not present any risk condition mentioned in stratum 1 and/or 2. The prevalence for each one of the distinct risk factor/condition, according to gender and age strata, is shown in Tables 1 and 2, respectively.

**Table 1:** Prevalence of major underlying conditions/risk factor for pneumococcal disease, according to gender, in people over 50 years in Catalonia, Spain

	MEN (*N* = 935,705) n (%)	WOMEN (*N* = 1,097,760) n (%)	ODDS RATIO (OR) OR 95%CI	OVERALL (*N* = 2,033,465) n (%)
Immunocompromising conditions				
Primary immunodeficiency	237 (<0.1)	352 (<0.1)	0.79 (0.67–0.93)	589 (<0.1)
HIV infection	2533 (0.3)	741 (0.1)	4.02 (3.70–4.37)	3274 (0.2)
Nephrotic syndrome	900 (0.1)	580 (0.1)	1.82 (1.64–2.02)	1480 (0.1)
Severe renal failure (GFR < 30)	6106 (0.7)	9362 (0.9)	0.76 (0.74–0.79)	15,468 (0.8)
Bone marrow trasplantation	3096 (0.3)	1938 (0.2)	1.88 (1.77–1.99)	5034 (0.2)
Recent cancer	55,475 (5.9)	48,473 (4.4)	1.36 (1.35–1.38)	103,948 (5.1)
Immunosuppressive medication/Radiotherapy	29,408 (3.1)	42,632 (3.9)	0.80 (0.79–0.82)	72,040 (3.5)
Other high risk conditions				
Asplenia	141 (<0.1)	153 (<0.1)	1.08 (0.85–1.37)	294 (<0.1)
Cochlear implant	27 (<0.1)	49 (<0.1)	0.65 (0.39–1.07)	76 (<0.1)
CSF leaks	17 (<0.1)	24 (<0.1)	0.85 (0.43–1.61)	41 (<0.1)
Chronic respiratory disease	115,432 (12.3)	96,823 (8.8)	1.45 (1.44–1.47)	212,255 (10.4)
Chronic heart disease	135,426 (14.5)	112,951 (10.3)	1.48 (1.46–1.49)	248,377 (12.2)
Chronic liver disease	23,290 (2.5)	18,444 (1.7)	1.49 (1.46–1.52)	41,734 (2.1)
Diabetes mellitus	183,649 (19.6)	157,886 (14.4)	1.45 (1.44–1.46)	341,535 (16.8)
Alcoholism	51,031 (5.5)	7750 (0.7)	8.11 (7.92–8.31)	58,781 (2.9)
Smoking	194,865 (20.8)	122,693 (11.2)	2.09 (2.07–2.10)	317,558 (15.6)

Odds Ratios (ORs) were calculated for men as compared with women. CI denotes confidence interval

**Table 2:** Prevalence of major underlying conditions/risk factor for pneumococcal disease according to age strata

	50–64 yrs. (*N* = 1,021,648) n (%)	≥ 65 yrs. (*N* = 1,011,817) N (%)	ODDS RATIO (OR) OR 95%CI
Immunocompromising conditions			
Primary immunodeficiency	295 (<0.1)	294 (<0.1)	1.01 (0.85–1.19)
HIV infection	2825 (0.3)	449 (<0.1)	0.16 (0.14–0.18)
Nephrotic syndrome	577 (0.1)	903 (0.1)	1.58 (1.42–1.76)
Severe renal failure (GFR < 30)	1058 (0.1)	14,410 (1.4)	13.94 (13.04–14.84)
Bone marrow trasplantation	2415 (0.2)	2619 (0.3)	1.10 (1.04–1.16)
Recent cancer	30,339 (3.0)	73,609 (7.3)	2.56 (2.53–2.68)
Immunosuppressive medication/Radiotherapy	22,319 (2.2)	49,721 (4.9)	2.31 (2.28–2.35)
Other high risk condition			
Asplenia	186 (<0.1)	108 (<0.1)	0.59 (0.46–0.75)
Cochlear implant	23 (<0.1)	53 (<0.1)	2.33 (1.39–3.91)
CSF leaks	11 (<0.1)	30 (<0.1)	2.75 (1.33–5.83)
Chronic respiratory disease	67,987 (6.7)	144,268 (14.3)	2.33 (2.31–2.36)
Chronic heart disease	49,362 (4.8)	199,015 (19.7)	4.82 (4.77–4.87)
Liver disease	20,567 (2.0)	21,167 (2.1)	1.04 (1.02–1.06)
Diabetes mellitus	100,855 (9.9)	240,680 (23.8)	2.85 (2.83–2.87)
Alcoholism	36,808 (3.6)	21,973 (2.2)	0.59 (0.58–0.60)
Smoking	242,626 (23.7)	74,932 (7.4)	0.26 (0.25–0.27)

Odds Ratios (ORs) were calculated for people ≥65 years as compared with people 50–64 years

CI denotes confidence interval

## Discussion

Pneumococcal disease is recognized as a major cause of morbidity and mortality around the world. [3] However, updated population-based data about the prevalence of some underlying medical conditions related to increasing risk of pneumococcal infections is limited. [8–11] We conducted a large population-based cross-sectional study assessing the prevalence of distinct risk underlying conditions to suffer pneumococcal diseases among 2,033,465 middle aged and older adults in a well defined geographical area in North-eastern Spain (Catalonia) with an overall population of seven million all-age inhabitants. The large size of the study population conforms an adequate basis to estimate population-based prevalence of these risk conditions in the Spanish population (which does not substantially differ from the Catalonian population). [22].

As main findings, approximately 10 % (8.7%) of the overall study population have some underlying conditions related with highest risk for pneumococcal disease (basically immunocompromising conditions) and 40 % (39.5%) have other conditions related to increasing risk (basically chronic pulmonary or cardiac disease, diabetes and/or smoking). Therefore, almost 50 % (48.2%) of the overall population may be considered at-risk for pneumococcal infections in our study. In the present study, most underlying conditions were more common in men than in women. Substantial differences (more than two-times higher prevalence in men than in women) were observed for smoking, alcoholism and HIV infection. Only prevalence of primary immunodeficiency, immunosuppressive treatment, severe renal failure, CSF leaks and cochlear implant were more frequent in women. Recent literature suggests that pneumococcal vaccine effectiveness may be influenced by sex, [24] which underlines the importance of gender as a stratification factor to assess epidemiological data and vaccine’s effectiveness.

Several studies have estimated the incidence of IPD among persons with high-risk conditions such as sickle-cell anemia, HIV infection, chronic pulmonary diseases, cardiac diseases, alcoholism and/or smoking [4–11, 25, 26] According to systematic reviews, [4, 7] anatomic or functional asplenia, immunodeficiency and immunocompromising conditions are considered as major risk factors for pneumococcal infections. Nevertheless, considering the relatively small prevalence of these high-risk conditions, the majority of IPD cases occur among immunocompetent subjects who have other conditions that are also associated with an increased risk of pneumococcal infections (e.g. chronic pulmonary or heart disease, diabetes mellitus, smoking). [4, 8, 9, 25–27].

Most studies assessing the presence of risk conditions for pneumococcal diseases were hospital based studies involving hospitalized patients with IPD, but there is limited population-based data about the prevalence of some of these conditions. [8–11] The accurate knowledge of this data is important to determine the true size of potential target populations for distinct antipneumococcal vaccination strategies. According to current recommendations of the *Advisory Committee on Immunization Practices* (ACIP) of the *Centers for Diseases Control and Prevention* (CDC, Atlanta, Ga, USA), a sequential pneumococcal vaccination using PCV13 + PPV23 is recommended for persons aged 65 years or older (with or without underlying conditions) and persons 19–64 years with CSF leaks, cochlear implants, functional or anatomic asplenia and/or immunocompromising conditions (including congenital or acquired immunodeficiency, human immunodeficiency virus infection, chronic renal failure, nephrotic syndrome, leukemia, lymphoma, Hodgkin disease, generalized malignancy, iatrogenic immunosuppression due to treatment with immunosuppressive medication or radiation therapy, solid organ transplant and multiple myeloma). [19, 20] At present, ACIP/CDC (Atlanta, Ga, USA) recommends pneumococcal vaccination using exclusively PPV23 for immunocompetent persons 19–64 years with other comorbidities or risk conditions such as pulmonary or cardiac disease, diabetes mellitus, alcoholism and smoking, [18] however, other vaccination guidelines recommend using PCV13 in these subjects. [15, 28] In Catalonia and Spain, antipneumococcal vaccination recommendations for adult basically agree with the ACIP’s recommendations except for people ≥65 years (where sequential PCV13 + PPV23 is only recommended for immunocompromised subjects). [13, 14].

In the present study, risk strata were defined on the basis of immunocompromise degree and presence of other underlying conditions generally considered to establish pneumococcal vaccination recommendations. According to the current ACIP’s recommendations (CDC, Atlanta, GA, USA), [18–20] all persons with any condition classified in our study as risk stratum 1 (basically immunocompromising conditions) should be vaccinated using a sequential PCV13 + PPV23 vaccination, whereas those persons classified as risk stratum 2 in our study (immunocompetent persons with other risk conditions such as chronic pulmonary or heart disease, diabetes, alcoholism or smoking) should receive PPV23 alone. Considering risk stratum 3 (immunocompetent persons without risk conditions), ACIP/CDC (Atlanta, GA, USA) recommends PCV13 + PPV23 for all people ≥65 years (with or without risk conditions) [20] whereas other experts and guidelines (e.g, Catalonian Health Service) recommends only PPV23 if they do not have chronic illnesses or risk conditions. [13, 29].

Our data provides important information for policy makers in order to better determine the size of different target population considering distinct pneumococcal vaccination strategies (e.g., recommendation of PCV13 for all subjects with any risk condition or only for those with immunocompromising conditions; sequential vaccination with PCV13 + PPV23 for all people >65 years or only for those with certain risk conditions; etc). According to our data, approximately 5% of people 50–64 years are at highest-risk for pneumococcal infections and consequently dual vaccination could be recommended. Within this 50–64 years age subgroup, approximately 37% of persons have other risk conditions and therefore should be vaccinated with a unique pneumococcal vaccine (PPV23 according to current ACIP/CDC recommendations). [18] If we consider elderly people (i.e. 65 years or older), almost half of them (45.3%) do not have underlying medical conditions requiring vaccination and they have only age criteria for increasing risk of pneumococcal infections. Thus a recommendation for dual vaccination based exclusively on age criteria >65 years (e.g. current ACIP/CDC recommendations) might be inefficient. [29].

In England, a study focused on adults who had received PPV23 estimated that 12.7% of population in 2009 had at least one of the risk conditions included in the recommendations for pneumococcal vaccination there (chronic pulmonary, cardiac, renal or liver disease, diabetes mellitus, immunocompromise, aspleny, cochlear implants and CSF leaks). [10] In Spain, a study conducted in Navarre estimated that almost 27% of the adult population had one or more risk condition of suffering pneumococcal disease. [30] In our setting, a prior cohort study involving 11,241 elderly individuals reported that 50.1% study subjects had no risk conditions for pneumococcal disease (apart from age), 29.2% had one risk condition and 20.7% had two or more risk conditions. [31] Methodological differences in outcome definitions, together with age differences in the study populations, may explain differences in the reported prevalence.

Our study has several strengths. Study design was population-based and large enough to assess the prevalence of main underlying risk conditions related to invasive pneumococcal infections. In the study setting, similar to the rest of Spain, all inhabitants are covered by the National Health Service by a compulsory health assurance system, all inhabitants are assigned to a PHCC, medical assistance is free and most treatments are publicly funded; so a bias related to people who are not seeking care or have not been entered into the SIDIAP database is unlikely. As limitation, although the validity of clinical data source was previously checked, [23] information bias may have occurred if some comorbidities or underlying conditions were not recorded. We do not have available data about the prevalence of some high-risk conditions (e.g. solid organ transplantations); so the true size of the high-risk stratum for pneumococcal infections may have been slightly underestimated.

Considering the epidemiology of pneumococcal disease most studies report specific data on elderly people (i.e, 65 years or older) who experience substantial morbidity and mortality. However, data focused on younger and/or middle-aged adults is uncommon. In the present study, together with elderly people, we also included middle aged persons (i.e, persons aged 50–64 years) because they have a non-insignificant incidence of invasive pneumococcal disease (around 10 episodes per 100,000 population year in developed countries), [8–11] and this was the cut off point (50 years or older) to recommend PCV13 when it was initially licensed for use in adults. [12].

## Conclusions

This large population-based study involving more than 2 million people investigating the prevalence of main risk conditions for pneumococcal infections among the general population 50 years or older in Catalonia, Spain, shows that approximately 50 % of them may be considered at-risk population for pneumococcal disease. Of this 50 % nearly 10 % have high-risk conditions and 40 % have medium-risk conditions. Updated epidemiological studies focused on distinct populations and geographical settings are necessary to know the prevalence of different risk conditions for pneumococcal disease, to clarify the true size of target populations for vaccination, and better allocate health care resources considering distinct vaccination strategies in adults.
